# Modified deep anterior lamellar dissection for corneal opacity during vitrectomy: case reports

**DOI:** 10.1186/s12886-020-01587-7

**Published:** 2020-08-03

**Authors:** Fang Li, Leilei Zhang, Yixiong Zhou, Dongqing Zhu

**Affiliations:** 1 Department of Ophthalmology, Ninth People’s Hospital, Shanghai JiaoTong University School of Medicine, Shanghai, 200011 China; 2 Shanghai Key Laboratory of Orbital Diseases and Ocular Oncology, Shanghai, China

**Keywords:** Corneal opacity, Deep lamellar corneal dissection, Retinal detachment, Vitrectomy

## Abstract

**Background:**

To introduce a modified deep anterior lamellar dissection technique to improve visibility during surgery for vitreoretinal diseases with coexisting corneal opacity.

**Case presentation:**

Two patients with retinal detachment and coexisting corneal blood staining or corneal decompensation underwent modified deep anterior lamellar dissections followed by vitrectomy. The modified deep anterior lamellar dissection techniques, unlike the dissection and removal of corneal lamellar in a typical deep anterior lamellar keratoplasty, included the creation and preservation of a deep lamellar corneal flap, the retroillumination to visualize and easily remove the remaining opaque stroma on the Descemet membrane, and the big air bubble technique in the eye with endothelial decompensation. The patient’s own cornea flap was sutured back after vitrectomy was done. The modified dissection techniques provided adequate fundus view during vitrectomy while removing as less corneal tissue as possible and decreasing the surgical complications and the requirement of a fresh cornea. Postoperatively, in case 1, the corneal blood staining was gradually absorbed and the vision improved from light perception to counting fingers. In case 2, even though the cornea remained cloudy and the vision was poor, the cornea endothelial decompensation was stable and asymptomatic. Both retinas were attached after silicone oil removal at 6-month follow-up.

**Conclusions:**

This modified and limited deep anterior lamellar corneal dissection procedure appears to be a useful alternative to penetrating keratoplasty, ophthalmic endoscope and temporary keratoprosthesis during the vitrectomy with coexisting corneal opacity.

## Background

Vitreoretinal surgery in the patients with coexisting corneal opacity is a challenge for retinal surgeons. For cases with dense corneal opacification which impedes the visualization for vitreoretinal intervention, the main solutions include traditional use of penetrating keratoplasty (PKP), ophthalmic endoscope and temporary keratoprosthesis (TKP) [1–4], or recent deep anterior lamellar keratoplasty (DALK) on only a few occasions [5, 6]. The main advantages of DALK are that it decreases the risks of endothelial rejection and decompensation, and eliminates the complications associated with open-sky procedure [7]. This method was first used in a case with hematocornea and posttraumatic retinal detachment in 2001 [5], and then applied to one patient with bullous keratopathy and retinal detachment in 2012 [6], In this technique, the opaque deep corneal lamella was removed before vitrectomy and replaced with a fresh corneal lamella at the end of surgery.

Recently, in two similar patients, we successfully performed modified and limited deep anterior lamellar corneal dissections to improve visibility during vitrectomy, which means removing as less corneal tissue as possible without requirement of a fresh cornea. The written informed consent for patient information and images to be published were obtained from the patients.

## Case presentation

### Case 1

**Fig. 1:**
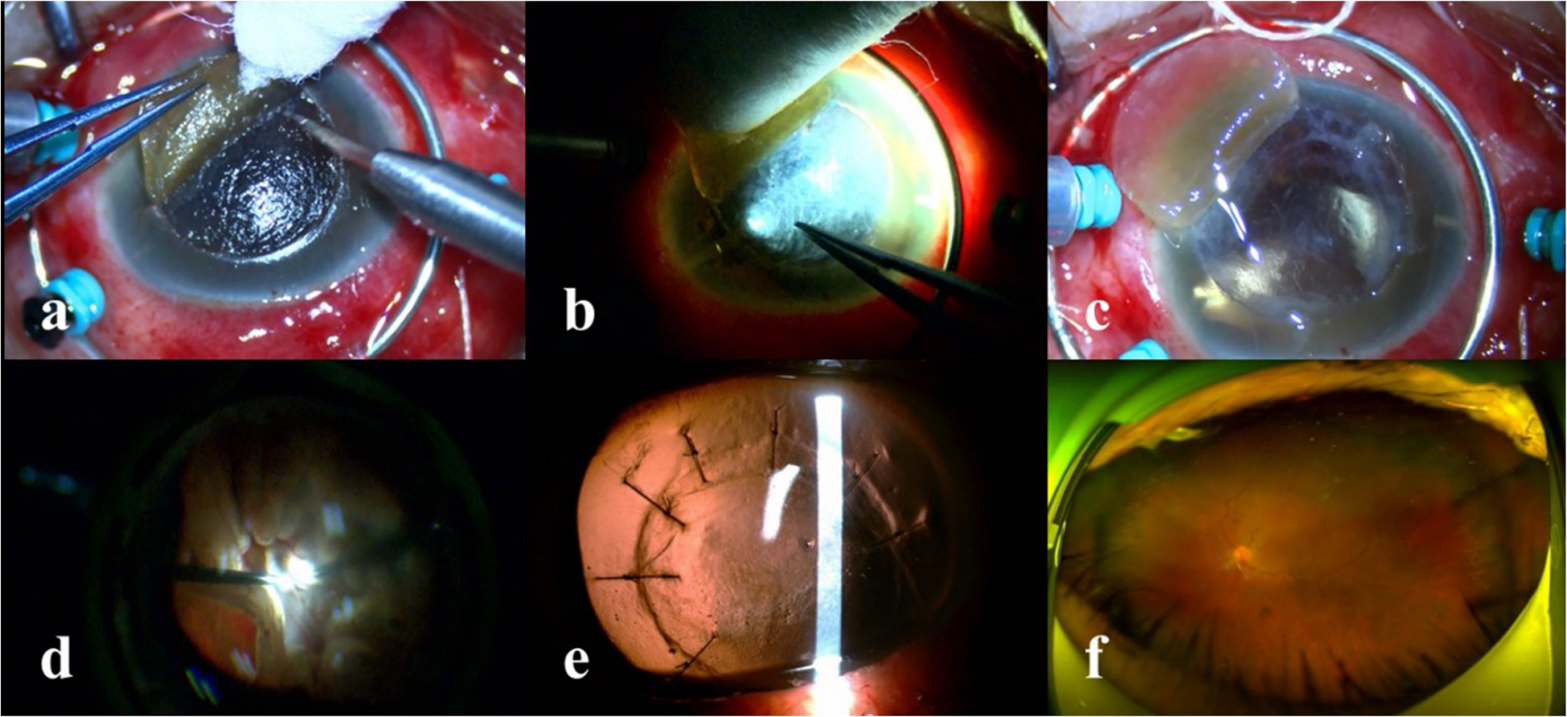
The modified corneal dissection in the case of corneal blood staining with coexisting retinal detachment. **a** A deep anterior lamellar corneal flap was created. **b** The remaining opaque deep stroma was peeled off with the help of retroillumination by an optic fiber through the peripheral anterior chamber. **c** The deep corneal bed provided good view of the intraocular structure. **d** Total retinal detachment could be clearly seen. **e** The cornea gradually became transparent within 3 months after the surgery. **f** Optomap image showed a flat retina after removal of silicone oil

A 62-year-old man presented with vision loss in his left eye after blunt trauma 2 weeks ago. He had undergone a discontinued PPV procedure because of inadequate fundus view at a local hospital 5 days ago. The vision in his left eye was light perception. Ocular examination showed conjunctival injection, complete hyphema associated with corneal blood staining. The intraocular pressure (IOP) was 11 mmHg. B-scan ultrasonography revealed retinal detachment and choroidal detachment. He was scheduled to undergo an urgent vitrectomy. After the hemorrhage in the anterior segment was cleared by irrigation, the lens was not seen, and the posterior segment could not be clearly viewed due to the corneal opacity. The technique of the modified deep anterior lamellar corneal dissection was performed. A shallow groove was gently made using a 7-mm trephine. Then a 15-degree slit knife was used to increase the incision depth and separate the deep anterior lamellar corneal tissue from the stroma anterior to the underlying Descemet membrane (DM) by cutting along the groove for about 3/4 circumference, leading to a corneal flap (Fig. 1a).The remaining deep stromal fibers were carefully peeled off using a forcep with the help of the retroillumination from a 25 gauge fiber optic illumination probe going through the peripheral anterior chamber (Fig. 1b). Finally, a moderately transparent corneal bed comprised of DM and a fine layer of deep stroma offered a clear enough view for pars plana vitrectomy (Fig. 1c). The dense vitreous hemorrhage was progressively removed, revealing a total retinal detachment in the shape of a closed funnel (Fig. 1d). Following the removal of epiretinal and subretinal proliferative fibrous membranes and peripheral retinotomy, the retina was reattached with application of perfluorocarbon liquid. Endolaser and silicone oil (Oxane 5700; Bausch & Lomb, Rochester, NY, US) tamponade were performed. Finally, the corneal flap was stitched back with interrupted 10–0 nylon (Video 1). Postoperatively, the cornea gradually became transparent, leading to a clear fundus view without impediment within 3 months (Fig. 1e). The silicone oil was removed 3 months after the surgery with a flat retina and improvement of vision to counting fingers (Fig. 1f). The IOP was 10 mmHg. The suture was removed 4 months postoperatively. The eye remained stable at the 6-month follow up.

**Additional file 1: Video S1.** The modified corneal dissection in the case of corneal blood staining associated with retinal detachment.

### Case 2

**Fig. 2:**
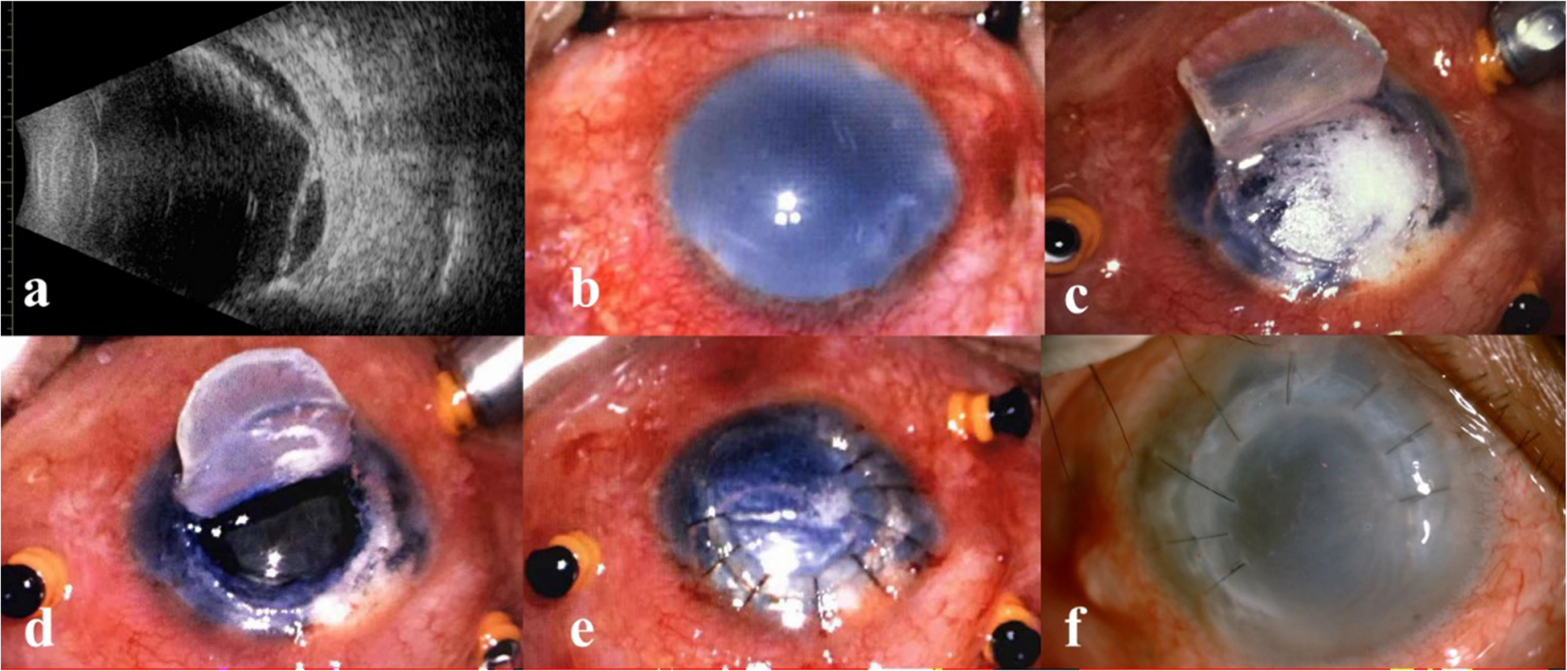
The modified corneal dissection in the case of corneal decompensation with coexisting retinal detachment. **a** B-scan ultrasonography revealed the retinal detachment. **b** Preoperative photo showed a cloudy cornea. **c** The big bubble technique was performed under the corneal flap. **d** Exposure of the smooth and clear Descemet membrane. **e** The corneal flaps were sutured back following vitrectomy. **f** The cornea remained cloudy, stable and asymptomatic after removal of silicon oil within 4-month follow up

A 45-year-old man suffered a recurrent retinal detachment associated with corneal endothelial decompensation in the right eye from a cataract combined with complicated retinal detachment surgery and subsequent repeat vitrectomies and silicone oil tamponade for complicated retinal detachment within 2 years. On examination, the vision was light perception in his right aphakic eye. The corneal stroma was entirely edematous while the epithelium showed intact. B-scan ultrasonography revealed retinal detachment (Fig. 2a). Poor visibility through the cloudy cornea precluded fundus view (Fig. 2b). The deep anterior lamellar dissection was also performed before vitrectomy. After the separation of anterior lamella as described above, the remaining corneal tissue could not provide adequate view of fundus. We thought the dissection was not deep enough because of the relatively thicker cornea. Then, the big-bubble technique was used to separate the posterior stroma from the DM (Fig. 2c). The deeper lamellar flap was also created and preserved. With good visualization of the intraocular structure through the transparent DM (Fig. 2d), retinal repair was performed with the application of retinectomy. After retinal reattachment and silicone oil tamponade as case 1, the two layers of corneal flap were sutured back (Fig. 2e). The cornea remained cloudy, but the retina was confirmed as attached by ultrasonography postoperatively. Three months later, the original corneal flaps were dissected again and the silicone oil was removed after intraoperative reconfirmation of retinal attachment. The opaque corneal flaps were sutured back. The eye was stable and asymptomatic within 4-month follow up (Fig. 2f). The vision was light perception and the IOP was 24 mmHg. Because the improvement of vision wouldn’t be possible, further surgery of corneal transplantation was not considered.

## Discussion and conclusions

Early techniques of anterior lamellar keratoplasty involve layer-by-layer manual stromal dissection and produce poor visual outcomes due to irregularity of the dissected surfaces and scarring in the tissue interfaces. Recent advances in techniques, especially the “big air bubble” technique, render the exposure of smooth DM easier and safer and increasingly popularize the DALK procedure [8, 9]. Studies indicated that the visual outcomes associated with DALK were similar to that with PKP [10]. The disadvantages of the DALK procedure are novelty, complexity and difficulty and a long learning curve. Inadvertent DM perforation may occur during the dissection process, which requires conversion to PKP or TKP. Perforations occurred in approximately 10 to 30% of cases [11].

Retinal detachment should be treated with surgery without delay, even in the presence of opaque cornea which precludes adequate visualization of intraocular structures. TKP or PKP was the choice for most retinal surgeons despite the increased risks during and after surgery. The challenges of performing DALK for retinal surgeons in this situation include the typical lack of surgical training in corneal surgery, unavailability of a surgeon familiar with deep corneal lamellar dissection techniques and absence of an alternate fresh donor cornea on emergency.

On the other hand, report demonstrated leaving a fine layer of healthy stroma attached to DM during the DALK surgery does not compromise the visual acuity [12]. Ocular trauma or diseases that affect both the anterior and posterior segments of the eye usually produce severe damage to vision. It appears that the interface haze formation in cornea has minimal effect on eventual visual outcomes in these eyes. Intentional sparing of the deepest stromal layer may prevent puncturing of DM during deep dissection process [13]. Compared to the uncontrolled original manual dissection by spatula, several modifications of the technique have been described to visualize and estimate the thickness of the deep stroma. The primary method proposed to fill the anterior chamber with air, which created a mirror effect so that the distance between the bubble and the blade tip could be determined. However, the usually preexisting infusion of anterior chamber during the combined surgery limited its use. Also, the red reflex-guided big-bubble technique, as proposed by Scorcia Vincenzo et al., [14] cannot be carried out due to the opaque posterior segment during the combined surgery. A handheld slit beam, [15] used to identify the presence of a big bubble during DALK, and an intraoperative anterior segment OCT technique [16] were limited due to high cost and unavailability of the special devices. In contrast, the bright retroillumination from the illumination probe used in our present procedure enhanced visualization of the posterior corneal surface, and helped to determine the remaining opaque stroma on the DM. Furthermore, it was not subjected to the status of posterior segment. The opaque stroma was not peeled off until the adequate view for vitrectomy was achieved, thus decreasing the risk of DM perforation. We think it is also applicable to a typical DALK.

Corneal blood staining occurs frequently in severe ocular trauma. It often gradually fades over time and replacement with a fresh cornea is not necessary. In the case 1 who needed urgent vitrectomy, we made a deep anterior corneal flap, allowing the adequate fundus view during surgery. This flap was restored to original position at the end of surgery. This procedure preserved the own corneal tissue, reduced the astigmatism and graft rejection, offered superior wound strength and early suture removal, and added no additional cost.

To minimize the scar formation between the anterior corneal flap and posterior corneal bed, an important step is the assessment of incision depth, which should be as close as possible to the DM, but without perforation of DM. In a typical DALK, the initial groove depth made by a trephine can be planed according to corneal biometry before surgery. However, to create a corneal flap, a very shallow groove should be made to outline the incision size. Careful incision along the groove and experience were required to increase and judge the depth. The direct visualization of dark and clear tissue or retroillumination may contribute to the accomplishment. In an edematous and cloudy cornea, a second incision and dissection can be performed, or following air bubble dissection, in order to preserve an intact DM.

Endothelial dysfunction is an indication for PKP or endothelial transplantation and considered as an absolute contraindication for DALK [17]. However, for the eyes associated with retinal detachment, the appropriate strategy is to perform an emergent vitrectomy firstly and then an elective corneal transplantation follows after retinal reattachment due to the lower success rate of corneal transplantation combined with vitrectomy. Successful DALK combined with vitrectomy was reported in previous case with corneal decompensation and retinal detachment [6]. A similar result was demonstrated in our case 2. Unlike the manual dissection they used, we attempted the big air bubble technique, and successfully separated the deep stroma from the DM, which proved that the air bubble technique was feasible in the condition of endothelial decompensation. This air bubble dissection was not different from the typical technique, except for the preservation of the second flap. The two dissections and flaps in this case not only provided clear fundus image but also created scar within corneal stroma which may prevent the epithelial edema and irritation.

In summary, our modified and limited deep lamellar cornea dissection preserved most of patients’ own cornea, prevented the perforation of DM, decreased the chance of graft failure and improved the overall visual prognosis while providing an adequate view for vitrectomy, which appears to be a useful alternative in the surgical treatment of patients with corneal opacity and vitreoretinal diseases.

## Data Availability

All data generated or analyzed during this study are included in this published article.

## Abbreviations

DALK: Deep anterior lamellar keratoplasty
DM: Descemet membrane
